# Symbiosis and microbiome flexibility in calcifying benthic foraminifera of the Great Barrier Reef

**DOI:** 10.1186/s40168-017-0257-7

**Published:** 2017-03-23

**Authors:** Martina Prazeres, Tracy Ainsworth, T. Edward Roberts, John M. Pandolfi, William Leggat

**Affiliations:** 1 College of Public Health, Medical and Veterinary Sciences, James Cook University, Townsville, QLD 4811 Australia; 2 ARC Centre of Excellence for Coral Reef Studies, James Cook University, Townsville, QLD 4811 Australia; 3 ARC Centre of Excellence for Coral Reef Studies and School of Biological Sciences, The University of Queensland, St. Lucia, QLD 4072 Australia

**Keywords:** Bacterial community, Large benthic foraminifera, *Amphistegina*, Photosymbionts, Environmental gradient

## Abstract

**Background:**

Symbiosis is a phenomenon that allows organisms to colonise a wide range of environments and occupy a variety of ecological niches in marine environments. Large benthic foraminifera (LBF) are crucial marine calcifiers that rely on photo-endosymbionts for growth and calcification, yet the influence of environmental conditions in shaping their interactions with prokaryotic and eukaryotic associates is poorly known.

**Results:**

Here, we used next-generation sequencing to identify eukaryotic photosynthesizing and prokaryotic microbes associated with the common LBF *Amphistegina lobifera* across a physio-chemical gradient on the Great Barrier Reef (GBR). We collected samples from three reef sites located in the inner-, mid- and outer-shelf regions of the northern section of the GBR. Results showed the consistent presence of Bacillaryophyta as the main eukaryotic taxa associated with *A. lobifera* across all reef sites analysed; however, the abundance and the diversity of prokaryotic organisms varied among reef sites. Inner-shelf specimens showed the highest diversity of prokaryote associates, with a total of 231 genotypes in their core microbiome. A total of 30 taxa were identified in the core microbiome across all reef sites. Within these taxa, *Proteobacteria* was the most abundant bacteria present. The presence of groups such as *Actinobacteria* was significantly correlated with inner-shelf populations, whereas the abundance of *Bacteroidetes* and *Firmicutes* was associated with *A. lobifera* collected from mid- and outer-shelf reef sites.

**Conclusions:**

We found that benthic foraminifera form stable and persistent symbiosis with eukaryotic partners, but flexible and site-specific associations with prokaryotic microbes that likely influence the ecological success of these crucial calcifying organisms on the GBR.

**Electronic supplementary material:**

The online version of this article (doi:10.1186/s40168-017-0257-7) contains supplementary material, which is available to authorized users.

## Background

Symbiotic associations expand both the diversity of potential ecological niches, and the metabolic capabilities of the host-symbiont partnerships [1]. Bacterial symbiosis facilitates the success of species across a variety of environmental conditions, playing a fundamental role in the evolution and adaptive capacity of eukaryotic organisms [2, 3]. In marine environments, bacterial associations can benefit non-photosynthetic eukaryotic hosts in deep sea and anoxic habitats through chemosynthesis [1], or by photosynthesis-dependent nitrogen fixation on coral reef, where light is usually abundant [4], in addition to other processes such as antibiotic production [5]. In contrast, algal symbiosis is one of the major mechanisms that allows mixotrophic nutrition, which is particularly beneficial in nutrient-depleted environments [6, 7] and can enhance calcification of photosymbiont-bearing hosts, such as corals and foraminifera [8]. Symbiotic relationships with both prokaryotes and eukaryotes have influenced the evolution of a number of organisms, resulting in both a departure from free-living existence and sometimes very unique ecological strategies [9, 10].

Reef-dwelling large benthic foraminifera (LBF) are single-celled protists that build a calcium carbonate (CaCO_3_) test and harbour algae as photo-symbionts [8]. They are integral elements of tropical coastal ecosystems, not only as important biological components, but also as key producers of the geological substratum (i.e. reef structure and sediments) [8, 11]. LBF are important marine calcifiers because they comprise up to 80% of the global reef carbonate sediment budget [12]. Foraminifera are estimated to account for 10–15% of the accumulated sediment of the Great Barrier Reef (GBR) [13], and approximately 35% of the total carbonate production in reef cays [14], equivalent to 4.75 × 10^2^ g CaCO_3_ m^−2^ y^−1^. Diverse groups of modern LBF host a wide variety of endosymbiotic algal groups (diatoms, dinoflagellates, unicellular chlorophytes and unicellular rhodophytes) and cyanobacteria, so foraminifera are particularly favourable partners for the establishment of symbioses [11]. As is the case with many other marine organisms [15, 16], LBF rely on symbiosis with photosynthetic algae for growth and calcification [17], and over the course of their evolution have developed morphological traits to accommodate endosymbionts in their tests [11]. In addition to algal symbionts, LBF can also be associated with diverse microbial communities [18–20], although this diversity is largely undocumented for most species, and their contribution to the success of foraminifera in the benthos is unknown.

Changes in environmental conditions can cause a shift in the microbial and in photosymbiont communities of organisms, resulting in a loss of some specific taxa and appearance of novel groups [21–23]. Flexibility in host-symbiont associations can be advantageous when environmental conditions change and is particularly important in the context of climate change, as host organisms that are flexible are able to form new symbioses that can be beneficial under new biotic and abiotic regimes [24, 25]. For example, molecular studies showed that the same species of LBF can host an extraordinary diversity of photo-symbiont types, up to 20 species of symbionts at any given time [24, 25]. Within-population symbiont polymorphism and mixed infections may be a mechanism by which foraminifera survive environmental fluctuations over time and colonise a wide range of habitats [26, 27].

The adaptability and symbiont flexibility of LBF is truly remarkable; not only are they able to acquire new symbionts if conditions change [24], but they can also shift their life history strategy from asexual division towards an increased dependence on sexual generations, ensuring horizontal transmission of symbionts [28]. In horizontal transmission, the host may acquire genetically diverse symbionts well suited for any given environment [29], which can then be perpetuated via vertical transmission through asexual reproduction [28].

Environmental degradation of coral reef ecosystems and coral bleaching from ocean warming have sparked increasing interest in the adaptive value and stability of symbiotic relationships of many reef organisms important to these ecosystems [11, 30, 31]. Host-microbiome interactions and/or symbioses are potential mechanisms by some organisms, such as corals, are able to successfully occupy a broad range of reef habitats [32, 33]. Given the importance of LBF for the maintenance and health of reef ecosystems, understanding how different environmental conditions impact the interactions of foraminifera with both eukaryotic and prokaryotic associates, and how flexible these associations are, is crucial for assessing their capacity to adapt and acclimatise to new conditions. Currently, few studies have investigated the microbial communities associated with LBF, highlighting the lack of data on prokaryotic microbiome system in foraminifera. These studies explored changes in microbial communities under controlled laboratory conditions [18–20] or reported preliminary characterisations of the bacterial microbiome [18]. Moreover, despite a substantial body of work on the description of eukaryotic photo-symbiont in LBF, these studies are all based in morphological features of the algal symbiont [11, 24, 25]. The aim of our study was to utilise next-generation sequencing to characterise the eukaryotic and prokaryotic microbiome of *Amphistegina lobifera*, which is a common LBF species in reef environments [34, 35], collected from different reef sites along a natural cross-shelf gradient of temperature and nutrients in the GBR.

## Results

### Photosynthetic microbes associated with *A. lobifera*

**Fig. 1:**
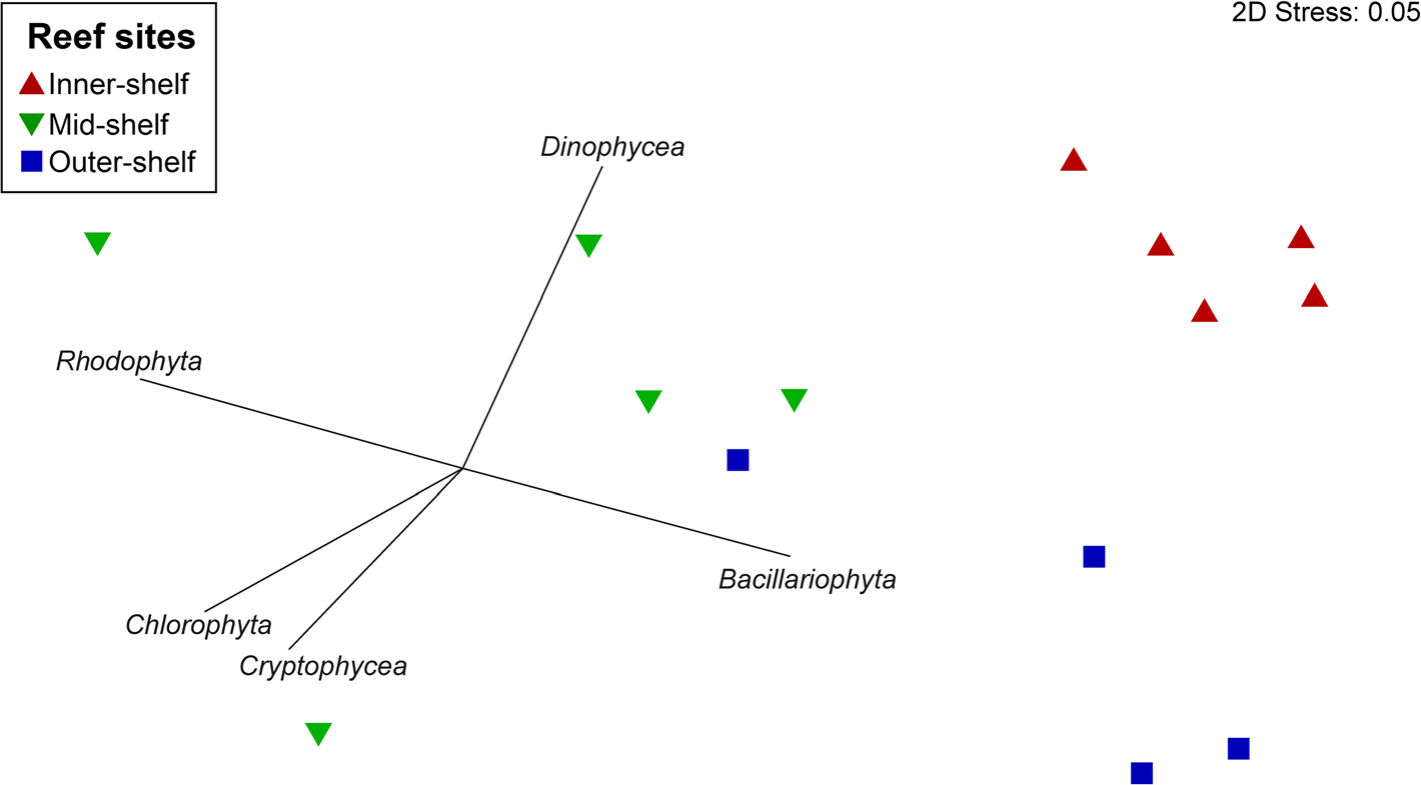
Differences in eukaryotic symbionts in *Amphistegina lobifera* collected from different reef sites. Two-dimensional non-metric multi-dimensional scaling plot with vectors displaying major algal groups identified in *A. lobifera*. Reef sites include inner-, mid- and outer-shelf locations of the northern GBR

The analysis of 18S rRNA of photosynthetic taxa sequences showed that specimens of *A. lobifera* host one dominant species of Bacillariophyta (OTU# denovo5251), which was consistently distributed across reef sites. Comparison of this OTU sequence using the Nucleotide BLAST of the National Centre for Biotechnology (NCBI) database showed 97% similarity to the diatom belonging to the order Fragilariales. Inner-, mid- and outer-shelf specimens of *A. lobifera* showed an average abundance of 96.06 ± 0.76%, 70.23 ± 5.89% and 81.71 ± 2.38% (mean ± SEM), respectively (Additional file 1: Table S1). Similar patterns were observed for 16S chloroplast rRNA sequences (OTU# 579531). Other algal taxa, such as Rhodophyta, were also detected in substantial proportion, and photosynthetic communities were different among sites (PERMANOVA: *n* = 15, F_(2, 14)_ = 2.65, *p* = 0.02; Fig. 1; Additional file 2: Table S2). Average relative abundance of Rhodophyta was as high as 16.63 ± 0.07% in mid-shelf samples. In addition to the dominant phylotypes, a total of 38 other OTUs were classified as Bacillariophyta (Additional file 3: Table S3). Diatom communities from inner-shelf *A. lobifera* differ from those found in the mid- and outer-shelf (PERMANOVA: *n* = 15, F_(2,14)_ = 11.95, *p* = 0.0001; Additional file 2: Table S4). Similarity percentage (SIMPER) analysis identified OTU# denovo7357 and OTU# denovo7981 as the main phylotypes driving the dissimilarity among samples (Table 1).

**Table 1:** Percentage (%) of contribution of Bacillariophyta taxa that primarily accounted for differences in *A. lobifera* collected from different reef sites. Percentage of contribution was calculated using the SIMPER analysis

Bacillariophyta OTU#	Inner-shelf × mid-shelf	Inner-shelf × outer-shelf	Mid-shelf × outer-shelf
Denovo2845	4.30	3.57	2.63
Denovo4054	3.43	5.17	2.59
Denovo7357	13.62	13.84	0
Denovo7981	4.27	1.66	7.78
Denovo10337	6.18	3.56	3.80

**Fig. 2:**
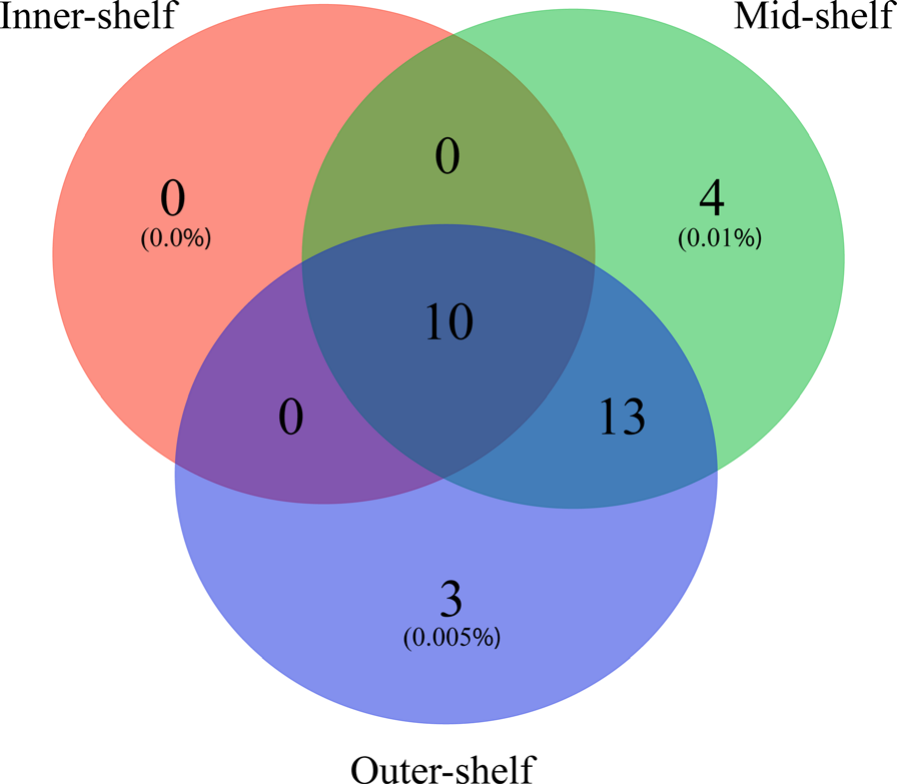
Venn diagram of 100% core diatom biome composition in *Amphistegina lobifera. Number* in brackets represents the relative contribution of core operational taxonomy units (OTUs) in relation to the total number of OTUs identified in each population of *A. lobifera*

Comparison of 18S rRNA sequences using the NCBI BLAST database identified these two OTUs as belonging to the orders Naviculales and Bacillarialles, respectively, as endosymbionts present in *A. lobifera* specimens collected from the mid- and outer-shelf. Cores from the mid- and outer-shelf consisted of more diatom OTUs than those from the inner-shelf. Ten OTUs were ubiquitous (Fig. 2) and comprised on average > 75% of the total OTUs classified as ‘Bacillariophyta’ (Additional file 3: Table S3). An additional 13 OTUs were found in all mid- and outer-shelf samples, while four (0.01%) and three (0.005%) OTUs were ubiquitous in only the mid- and outer-shelf samples, respectively. In contrast, no OTUs were exclusively found in the inner-shelf (Fig. 2).

### Microbiome of *A. lobifera* collected from different reef sites

**Fig. 3:**
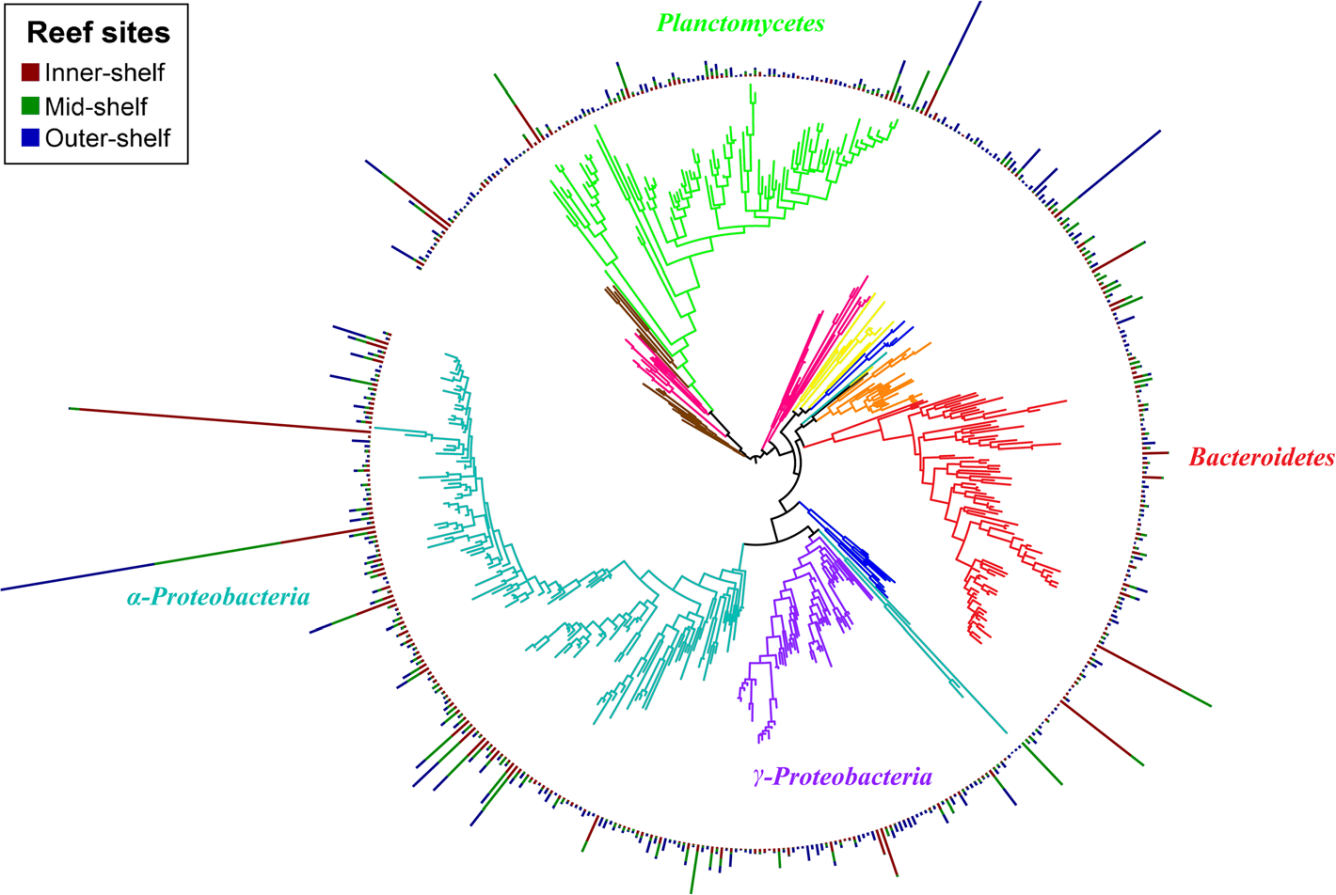
Phylogenetic tree of microbial community associated with *Amphistegina lobifera. Dendrogram* represents the 451 bacterial operational taxonomic units (OTUs) with a high number of reads (≥100 counts) across all samples. The most diverse and abundant taxa are *highlighted. Bars* represent relative abundance of each OTU identified in *A. lobifera* collected from different reef sites

**Fig. 4:**
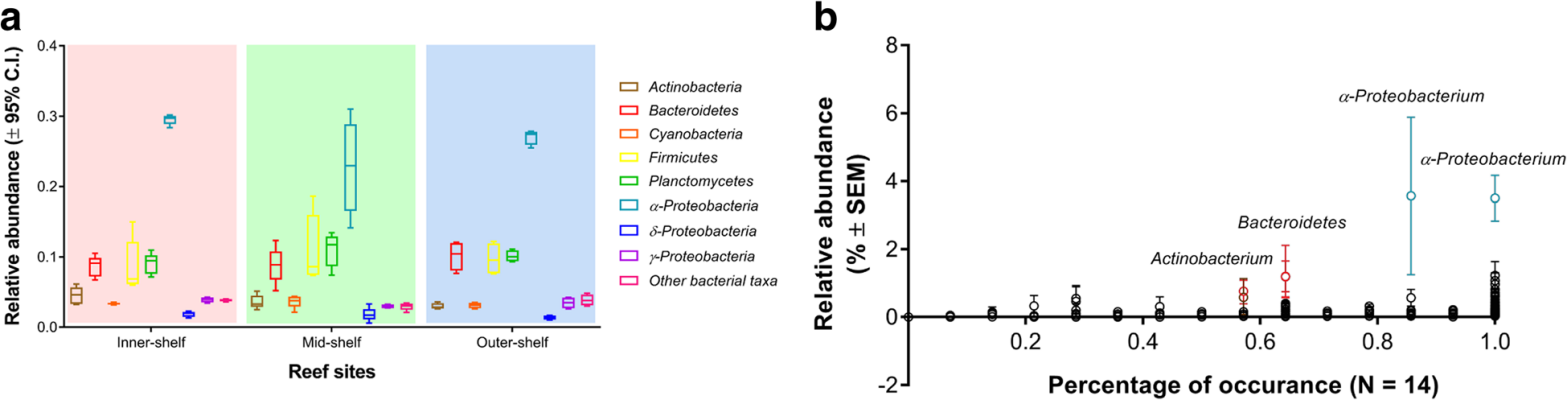
Bacterial community identified in *A. lobifera* across different reef sites. **a** Relative abundance of major bacterial groups in *Amphistegina lobifera* (mean ± 95% C.I, *n* = 14) across reef sites studied. *Bars* represent 95% C.I. and *boxes* represent quartiles. **b** Comparison of relative abundances (mean ± SEM, *n* = 14) and percentages of occurrence of operational taxonomic unit (OTU) across all samples. Each *point* represents as individual OTU

**Fig. 5:**
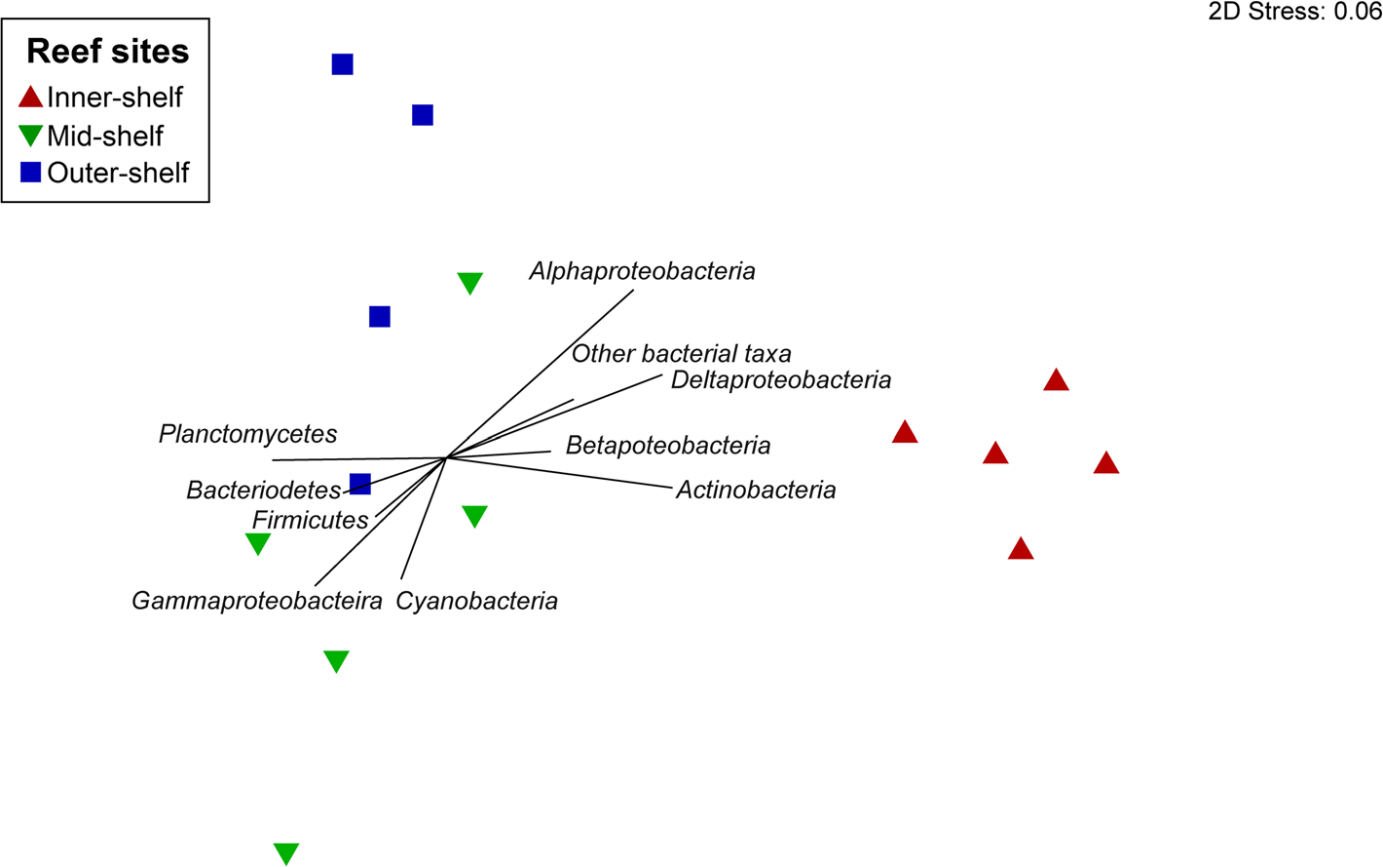
Differences in bacterial communities in *Amphistegina lobifera* collect from different reef sites. Two-dimensional non-metric multidimensional scaling plot with vectors showing major bacterial groups identified in *A. lobifera*. Reef sites include inner-, mid- and outer-shelf locations of the northern GBR

The microbial community of *A. lobifera* consisted of 13,218 identified OTUs. After the removal of singleton and low counts OTUs (>100 counts summed across all samples), a total of 451 OTUs remained, belonging mainly to the following bacterial taxa across all reef sites: *Proteobacteria* (31.57 ± 1.60%, mean ± SEM), *Planctomycetes* (10.04 ± 0.46%), and *Firmicutes* (9.85 ± 0.99%; Fig. 3). Among these OTUs, *α-Proteobacteria* were consistently the most abundant and diverse group of bacteria present across reef sites with an average abundance between 22.76 and 29.54% (Fig. 4a; Additional file 4: Table S5). *α-Proteobacteria* was also consistently found across all samples analysed (Fig. 4b). There was a distinct difference in the microbial community of *A. lobifera* among reef sites (PERMANOVA: *n* = 14, F_(2,13)_ = 2.23, *p* = 0.0002; Fig. 5). Pair-wise comparison showed a significant difference between the inner-, mid- and outer-shelf microbial communities (Additional file 2: Table S6). SIMPER analysis showed that the dissimilarity between *A. lobifera* collected from the inner-shelf and both mid- and outer-shelf sites was due to the presence of a different lower raking taxa within *Firmicutes*, *Actinobacteria* and *Bacteroidetes*, whereas the dissimilarity between mid- and outer-shelf sites was mainly due to the presence of different *Firmicutes* and *γ-Proteobacteria* taxa (Table 2). Total richness estimators showed that bacterial diversity was similar among reef sites (Additional file 5: Figure S1, Additional file 2: Table S7). The clear distinction between inner-shelf and mid/outer-shelf samples was driven by the abundance of different bacterial taxa (Fig. 5). Taxa such as *δ-Proteobacteria* and *Actinobacteria* are positively correlated with inner-shelf samples, with *Firmicutes* and *γ-Proteobacteria* correlated with mid/outer-shelf samples. *Planctomycetes* and *Bacteroidetes* taxa are also associated with mid/outer-shelf samples, but not as strongly (Fig. 5).

**Table 2:** Percentage (%) of contribution of bacterial taxa that primarily accounted for differences in *A. lobifera* collected from different reef sites. Percentage of contribution was calculated using the SIMPER analysis

Bacterial taxa	Inner-shelf × mid-shelf	Inner-shelf × outer-shelf	Mid-shelf × outer-shelf
*α-Proteobacteria*	13.73	5.94	12.54
*β-Proteobacteria*	10.12	11.53	11.67
*δ-Proteobacteria*	7.12	8.41	0
*γ-Proteobacteria*	12.43	7.08	13.22
*Actinobacteria*	9.17	16.72	7.45
*Bacteroidetes*	8.51	12.10	10.76
*Cyanobacteria*	0	0	8.35
*Firmicutes*	15.75	18.97	13.39
Other bacterial groups	7.35	6.61	8.52
*Planctomycetes*	9.52	8.16	7.87

**Fig. 6:**
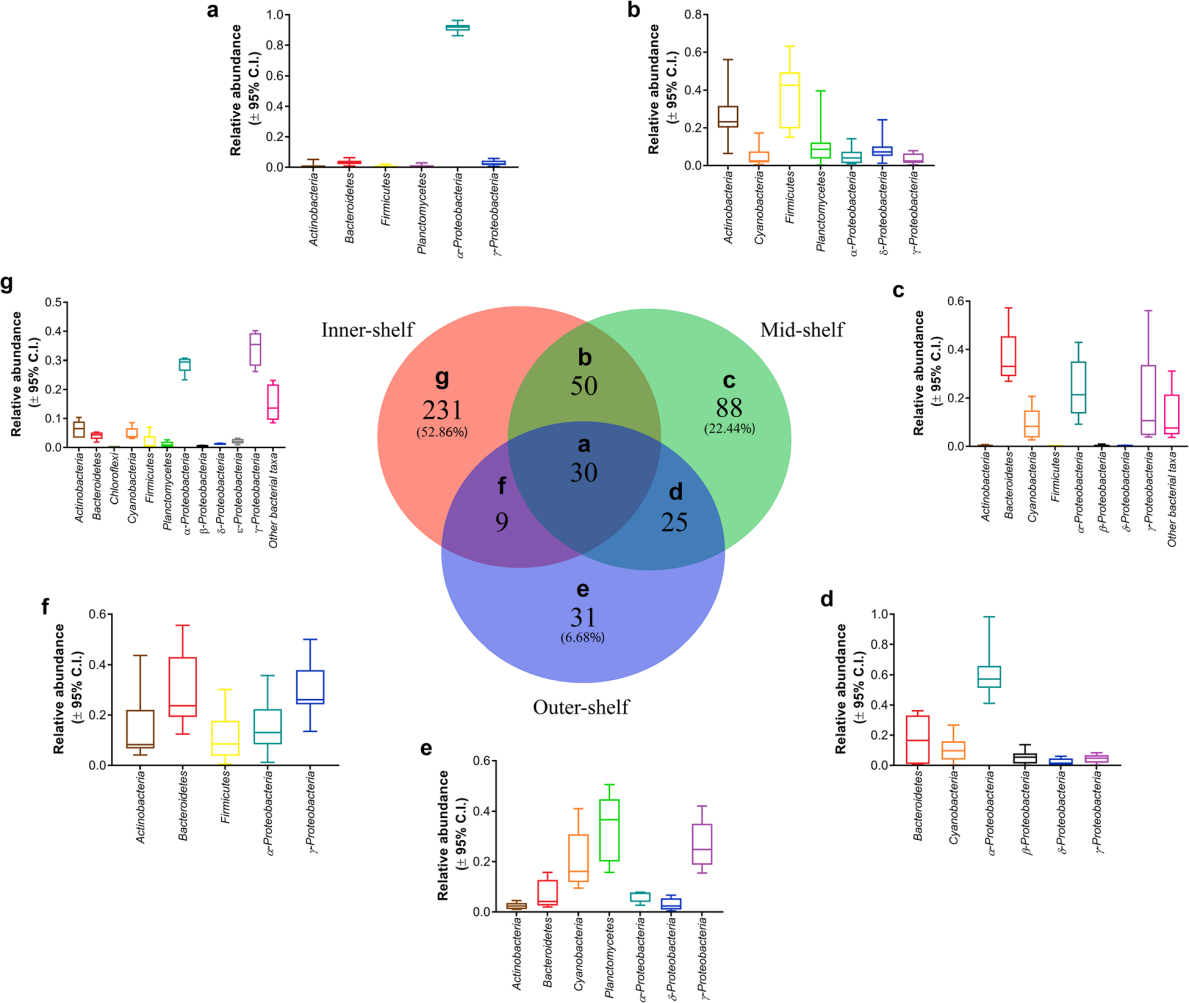
Venn diagram of 100% core microbiome in *Amphistegina lobifera. Boxplots* indicate the bacterial phylum/class and respective relative abundance (mean ± 95% C.I.) of the bacterial taxa identified in each core. *Number* in brackets represents the relative contribution of core operational taxonomy units (OTUs) in relation to the total number of OTUs identified in each population of *A. lobifera*. **a** Inner-, mid- and outer-shelf core microbiome. **b** Inner and mid-shelf core microbiome. **c** Mid-shelf core microbiome. **d** Mid- and outer-shelf core microbiome. **e** Outer-shelf core microbiome. (**f**) Inner- and outer-shelf core microbiome. **g** Inner-shelf core microbiome

Analysis of the core microbiome showed that *α-Proteobacteria* was the most consistent bacterial taxa found in *A. lobifera* across different reef sites. Among the 30 ubiquitous bacterial taxa identified (Fig. 6), 13 were classified as *α-Proteobacteria*. Other bacterial taxa included *Cyanobacteria* (5), *Actinobacteria* (4), *Firmicutes* (4), *Planctomycetes* (3) and *γ-Proteobacteria* (1). However, *Firmicutes* showed the highest average abundance in the core across all reef sites (Fig. 6b). Core microbiome among reef sites showed a different pattern of bacterial relative abundance and diversity. In contrast to the eukaryotic photo-autotrophs, *A. lobifera* from the inner-shelf site showed the highest diversity of bacterial taxa (231 OTUs found exclusively in the inner-shelf core), whereas the lowest diversity of bacterial taxa was found in inner- and outer-shelf samples. It is noteworthy that *Chloroflexi,* which was exclusively found within the inner-shelf, was present in all inner-shelf samples. Specimens collected from the mid-shelf showed a high average proportion of *Bacteroidetes*, while outer-shelf samples showed a high average proportion of *Planctomycetes*.

## Discussion

The dominant photo-symbiotic partners in large benthic foraminifera (LBF) are well known and described [11]; however, the distribution patterns of their bacterial associates are poorly understood. This study identified a small group of bacteria that are ubiquitous across three populations distributed broadly across the GBR shelf system and revealed that the common and abundant bacterial taxa associated with *A. lobifera* within each population drove differences in the community structure of bacteria at different reef locations. In contrast, the photo-symbiotic profile in *A. lobifera*, mainly the taxanomic composition of the rare diatoms, was site-specific. Thus, the most abundant photo-symbiont taxa in *A. lobifera* were highly conserved among reef sites, but bacterial communities were very flexible. Differences in environmental conditions, mainly between inner-shelf and mid/outer-shelf locations, are likely to have a major influence in shaping the bacterial communities associated with *A. lobifera* populations. On the GBR, factors such as nutrient concentration and temperature vary across an inshore-offshore gradient. Inshore reef sites are more prone to temperature fluctuations and influx of dissolved inorganic nutrients from terrestrial runoff, whereas offshore sites display more stable temperature conditions and low nutrient concentrations [36, 37]. The capacity to acquire different eukaryotic symbionts (i.e., photosynthetic algae) and prokaryotic associates is likely to be a potential driver of the ability of LBFs to occupy a broad range of habitats in reef environments.

Fragilariales was the most abundant and common order identified. Within this order, *Nanofrustulum shiloi* is known to be a common endosymbiont species in *A. lobifera* [25]. This phylotype of microalgae was ubiquitously found in high abundance across all three populations of *A. lobifera*, suggesting that *A. lobifera* associates with this species regardless of continental shelf location. Other diatom species were also identified, within the orders Naviculales and Bacillariales, although at lower densities (~5–10%, Additional file 2: Table S2). Both orders have been previously described as endosymbionts of *Amphistegina* sp. [24], further supporting the notion that individual *A. lobifera* can maintain partnership with multiple symbiont types [38]. Interestingly, *A. lobifera* collected from offshore reef sites showed a higher diversity of diatoms, suggesting that the clear, oligotrophic environment of the mid- and outer-shelf may allow for the colonisation of a broader array of photo-symbionts that benefit from more stable conditions of light, lower temperature fluctuations, and lower nutrients. In contrast, inner-shelf specimens may select strains of diatoms that can tolerate waters with low light levels, regular pulses of nutrients and peaks in elevated temperature. The presence of other algal groups such as Rhodophyta, Chlorophyta and Dinophycea sequences can be due to the common association of *A. lobifera* with reef rubble that is covered with turf algae [39]. Additionally, species of LBF can host a variety of algal groups as endosymbionts [10]. Even though uncommon, the same species of LBF is able to host photo-symbionts belonging to different major groups [11]. The presence of rhodophytes and dinoflagellates, for example, in *A. lobifera* samples collected from mid- and outer-shelf populations could be linked to the presence of these taxa as endosymbionts, although in very low relative abundances.

The high bacterial diversity among foraminifera species found in laboratory studies has been found to be a result of their close association with reef rubble, sediment and filamentous algae [18]. In the present study, groups such as *Proteobacteria* (mainly *Alpha*, *Delta* and *Gamma* classes), *Planctomycetes*, *Bacteroidetes* and *Firmicutes* were consistently the most abundant and diverse phyla of bacteria associated with *A. lobifera*. However, the classes and genera within these major phyla varied significantly across sites, indicating flexibility in prokaryotic association within *A. lobifera* from different habitats. Bacterial diversity in *A. lobifera* collected from the inner-shelf was the highest. It is not possible to determine the kind of interaction bacterial taxa have with *A. lobifera*; however, the consistent within and between site patterns observed in the bacterial associations indicate that bacterial communities are non-random. Uthicke and McGuire [40] argued that differences in water quality along a cross-shelf gradient could drive differences in bacterial communities associated with carbonate sediments. Similarly, the highest diversity of bacterial taxa we observed in inner-shelf samples may be associated with higher availability of organic matter in inshore reefs of the GBR due to terrestrial runoff [41], which benefits heterotrophic bacteria [42]. The mechanism that drives the cross-shelf gradient in carbonate sediments is likely to be similar to the one driving cross-shelf patterns in bacterial communities in *A. lobifera*. However, bacterial communities previously detected in sediment samples along a cross-shelf gradient [40] differ from those found in our study, indicating active interaction of bacterial taxa with *A. lobifera.*

Phyla such as *Actinobacteria*, which is commonly associated with freshwater runoff [41, 43], showed a strong positive correlation with *A. lobifera* collected from the inner-shelf reef site. *Actinobacteria* is also known to be a common invertebrate symbiont, commonly associated with zooxanthallae corals and can be associated with marine sediments [33, 44]. This group of bacteria is reported to contribute to the breakdown and recycling of organic compounds [45], which can be particularly advantageous to hosts in habitats where the amount of organic matter associated with carbonate sediments is high. In contrast, the increased relative abundance and presence of *Cyanobacteria* associated with *A. lobifera* collected from mid/outer-shelf reef sites might be explained by higher light availability for this photo-autotrophic group of bacteria, which has a competitive advantage in oligotrophic environments [46]. *Cyanobacteria* also naturally occur as benign endosymbionts in some LBF species [26] and play a role in N-fixation within the host when conditions are optimum [4]. Although our study demonstrates the presence of a small group of bacteria (in total 30 OTUs identified) that are persistently associated with LBFs across reef sites, a large proportion of bacterial taxa identified by the core microbiome analysis are site-specific, and likely help *A. lobifera* to colonise a wide range of environmental conditions.

In summary, *A. lobifera* from the inner-shelf showed the lowest diversity of diatoms, and the highest diversity and abundance of prokaryotic symbionts. No diatom was exclusively found in *A. lobifera* from the inner-shelf. While the number of prokaryotic microbes shared between different *A. lobifera* populations was small, the core diatom biome was large, with the vast majority of diatoms conserved in all replicate individuals per site. In contrast, the core microbiome within each site, especially in inner- and mid-shelf specimens, was higher than the core shared by all three sites together. These results indicate that prokaryote microbes are likely to play a crucial role in the ecology of *A. lobifera*. Environmental variables such as water quality, temperature fluctuations and light exposure may help drive the compositional differences in the prokaryotic and eukaryotic microbial communities in *A. lobifera* from the GBR *A. lobifera* specimens collected from different habitats display substantially different responses when exposed to experimental conditions such as increased temperature and nitrate [47], as they have the capacity to assimilate different types of eukaryotic and prokaryotic associates according to their local habitat.

## Conclusions

We demonstrated that *A. lobifera* specimens are able to establish strong but not specific symbiosis with their eukaryotic endosymbionts. This species also showed a diverse and flexible interaction with bacterial associates, which varied among reef sites. This study, combined with previous work [47, 48], demonstrates how microbial communities can help shape the resilience and resistance of LBFs to changing environmental conditions such as climate change, and further highlights the importance of symbiotic relationships in their capacity to colonise a broad range of reef habitats. Whether the core microbiome is responding to or is being filtered by environmental gradients remains to be investigated. Given the importance of LBFs as ecosystem engineers and prolific carbonate producers [12], understanding the abiotic and biotic factors that control the diversity and associations of LBFs with microbe symbionts is crucial to assess their capacity to acclimate or adapt to global and local impacts.

## Methods

### Study sites and sampling collection

**Fig. 7:**
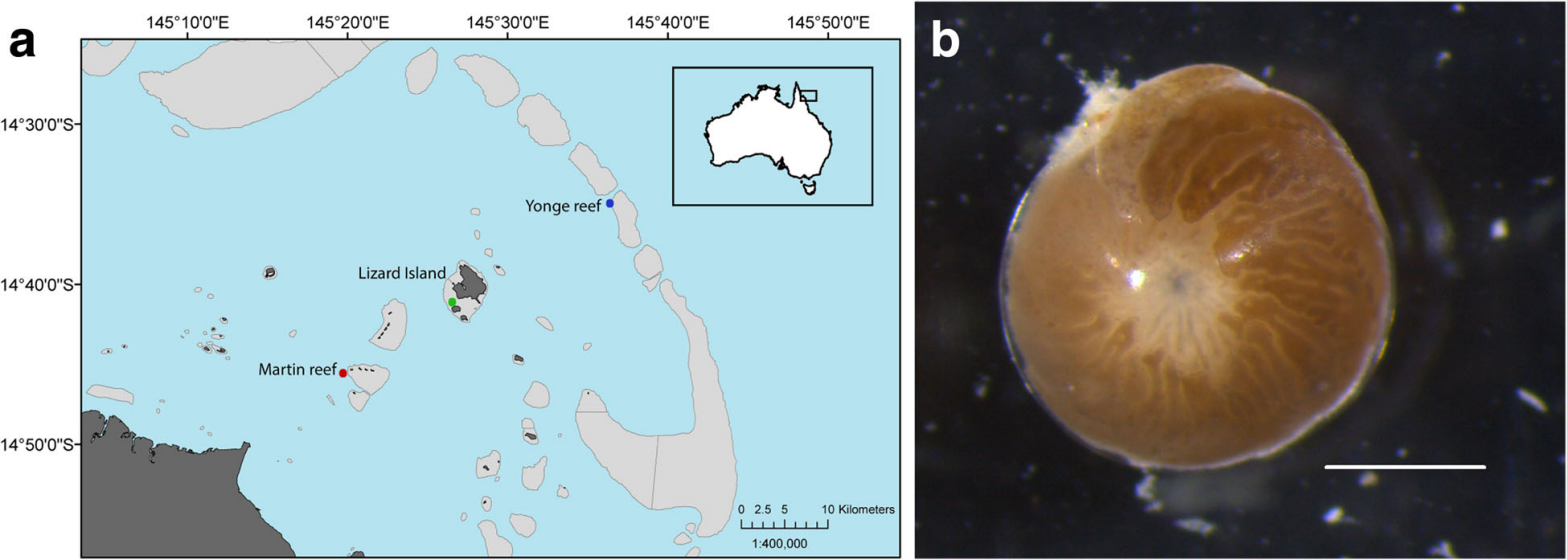
Location of reef sites and *Amphistegina lobifera* specimen. **a** Sampling sites across the continental shelf on the Far North section of the Great Barrier Reef, Australia. Inner-shelf reef: Martin reef; Mid-shelf reef: Lizard Island; Outer-shelf reef: Yonge reef. Map was generated using the software ArcGIS v10.2 (www.esri.com). **b** Image of *A. lobifera. Scale bar* = 0.5 mm. Photography by M.P

Dead coral rubble with *A. lobifera* attached was collected from inner-, mid- and outer-shelf reefs located on the northern GBR in August 2014 (Fig. 7). Samples were collected by SCUBA divers from the back slope of reefs with similar habitat located on the (1) inner-shelf—Martin Reef (14° 45′ 19.2″ S; 145° 20′ 07.9″ E), (2) mid-shelf—Lizard Island (14° 14′ 22.3″ S; 145° 27′ 58.1″ E), and (3) outer-shelf—Yonge Reef (14° 35′ 50.1″ S; 145°37' 26.3"E) at depths of 6 to 8 m (corrected to lowest astronomical tide levels). Reef sites are located along a water quality gradient [36, 49] and also experience differing patterns of temperature fluctuation [37, 47, 50]. Pieces of dead coral rubble were brought to the laboratory located at the Lizard Island Research Station and were scrubbed to remove LBF. The resultant sediment was transferred to glass Petri dishes and was placed undisturbed in a flow-through aquarium system. A total of five *A. lobifera* individuals per reef site were extracted and cleaned using a fine brush (#000) under a stereoscope to remove any particles from the test exterior and photographed. Specimens were then snap frozen in liquid N_2_ and kept at −80 °C until analysis.

### DNA extraction and sequencing

In the laboratory, specimens were taken from −80 °C, rinsed in cold sterile phosphate buffer saline 3× and immediately placed individually in 1.5-ml tubes for DNA extraction. Each tube contained 200 μl of lysis buffer (QIAmp® DNA Mini Extraction kit, Qiagen) containing Proteinase K and crushed using a micro homogenizer. Samples were incubated overnight at 56 °C and then were purified using a silica-membrane-based nucleic acid technique (QIAmp® DNA Mini Extraction kit, Qiagen). Extracted DNA concentration was quantified using a Qubit® High-sensitivity dsDNA assay kit (Life Technologies, NSW, Australia). Purified total DNA samples were sent to Molecular Research Laboratory (Shallowater, TX, USA) for the PCR amplification and Illumina sequencing of the rRNAs of eukaryotic and prokaryotic associates.

### PCR amplification, sequencing and sequence analyses

Bacterial 16S and eukaryotic 18S rRNA samples were PCR-amplified from the genomic DNA template and were sequenced using the Illumina HiSeq250 platform. PCR amplification was performed using the universal Eubacterial primers 27 F (5′ -AGAGTTTGATCCTGGCTCAG) and 519R (5′ GTNTTACNGCGGCKGCTG), which target highly variable regions V1, V2 and V3 of the 16S rRNA. We also targeted eukaryotic 18S rRNA using the following primer set: Euk1391 (5′ GTACACACCGCCCGTC) and EukBRev (5′ TGATCCTTCTGCAGGTTCACCTAC). A single-step 30-cycle PCR using HotStarTaq® Plus Master Mix Kit (Qiagen, Valencia, CA) was used under the following conditions: 94 °C for 3 min, followed by 28 cycles of 94 °C for 30 s; 53 °C for 40 s and 72 °C for 1 min; after which a final elongation step at 72 °C for 5 min was performed. Following PCR, all amplicon products from different samples were mixed in equal concentrations and were purified using Agencourt Ampure beads (Agencourt Bioscience Corporation, MA, USA). Samples were sequenced utilising Illumina HiSeq250 instruments and reagents (Shallowater, TX, USA). Negative controls for each amplification and sequencing stage were utilised. The sequence data were processed using the software package QIIME [51]. Raw .sff sequence reads from all samples were depleted of barcodes, then sequences <200 bp, with ambiguous base calls and homopolymer runs exceeding 6 bp were removed. Sequences were then denoised, demultiplexed, and chimeras removed using UCHIME [52]. 18S rRNA sequences were aligned and classified at 97% similarity using the SILVA 108 database [53]. Reads that were classified as ‘Fungi’, ‘Rhizaria’, ‘Metazoa’, ‘environmental samples’ or ‘unassigned’ were removed from the OTU table, and only photosynthetic taxa analysed. 16S rRNA sequences were aligned and operational taxonomy units (OTUs) were defined using RDP classifier at 97% similarity against the May 2013 curated GreenGenes database [54]. Any sequences that were classified as ‘chloroplast’ or ‘unassigned’ were filtered out of the dataset. Also, for further comparison with 18S rRNA results, only sequences classified as ‘chloroplast’ were retained into an additional database.

### Statistical analyses

Differences in eukaryotic and prokaryotic microbes associated with *A. lobifera* populations collected from differing reef sites were analysed using the software QIIME and PRIMER 6.1.15 with PERMANOVA+ 1.0.5 [55]. Prior to the analyses, singleton and ‘low read’ OTUs (<100 counts summed across all samples) were removed prior to normalisation. Additionally, for the 16S rDNA, sequence reads were normalised to 5775 reads per sample, as this was the lowest number of reads among all samples analysed, to allow for comparison between samples and bacterial community, which further excluded one sample from the outer-shelf reef site. For each OTU table, standardised relative abundances of each taxon were fourth root transformed to reduce the influence of rare and dominant taxa. Homogeneity of variance was confirmed for the factors ‘reef site’ using PERMDISP, a distance-based test for homogeneity of multivariate dispersions [55]. Differences within and between each reef site were analysed through a Permutation Multivariate Analysis of Variance ANOVA (PERMANOVA) using a Bray-Curtis resemblance matrix, employing ‘reef site’ as a fixed factor. PERMANOVA and subsequent pairwise tests were based on 9999 permutations, using type III sums of squares and permutation of residuals under an unrestricted model. For comparison, we also evaluated beta diversity using unweighted and weighted UniFrac [56] pipeline in QIIME for both 16S and 18S datasets. A two-dimensional non-metric multidimensional scaling (nMDS) ordination was used as a visual representation of the compositional differences among eukaryotic and prokaryotic microbes associated with foraminiferal populations from different reef sites. In each nMDS plot, we plotted main groups of bacterial or eukaryotic taxa as vectors to examine the groups that influenced patterns in differences among reef sites. Since *A. lobifera* is known to harbour diatoms, we filtered out the other algal groups from the 18S dataset and retained the OTUs classified as ‘Bacillariophyta’ in order to compare the composition of endosymbionts among reef sites using PERMANOVA. For both 16S and 18S datasets, a SIMPER analysis was performed on the fourth root transformed datasets to determine those microbial or eukaryotic groups that were responsible for the significant dissimilarity between *A. lobifera* specimens collected from different locations, as identified using pairwise comparisons [57]. SIMPER decomposes average Bray-Curtis dissimilarities between all pair of samples into percentage contributions from each OTU, listing the OTUs in decreasing order of contribution for dissimilarity [57]. For the bacterial dataset, a phylogenetic tree was constructed in QIIME and visualised using the online Interactive Tree of Life [58, 59]. The phylogenetic tree was based on the approximately maximum likelihood bootstrap of aligned 16S rRNA gene sequences using the FastTree program in QIIME [60]. Total richness estimating curves of the alpha diversity of bacterial taxa were also generated. Finally, in order to identify the stable, consistent diatom and bacterial taxa present in *A. lobifera* specimens collected from different reef sites, core microbiome and diatom biome were identified using the software package QIIME. Core microbiome and diatom biome were defined as the OTUs that were present in 100% of the samples across the reef sites. Venn diagrams were generated utilising the core diatom species composition and microbiome and constructed with the online Venn diagram software from Bioinformatics and Evolutionary Genomics (http://bioinformatics.psb.ugent.be/software/details/Venn-Diagrams).

## Additional files

Additional file 1: Table S1. Absolute and relative abundance of photosynthetic eukaryotes associted with *A. lobifera* collected from different reef sites. (XLS 75 kb)

Additional file 2: Table S2. Results of main effects PERMANOVA model of photosynthesizing eukaryotic taxa associated with *Amphistegina lobifera* collected from different reef sites. **Table S4.** Results of main effects PERMANOVA model and pairwise comparison of Bacillariophyta taxonomic composition in *Amphistegina lobifera* collected from different reef sites. **Table S6.** Results of main effects PERMANOVA model and pairwise comparison of bacterial community in *Amphistegina lobifera* collected from different reef sites. **Table S7.** Results of alpha diversity comparison between samples using a parametric *t* test using a *t* distribution. (DOC 58 kb)

Additional file 3: Table S3. Relative abundance of Bacillariophyta associated with *A. lobifera* across all reef sites. (XLS 39 kb)

Additional file 4: Table S5. Absolute and relative abundances of OTUs identified, and main bacterial taxa associated with *A. lobifera* across all reef sites. (XLS 213 kb)

Additional file 5: Figure S1. Species richeness estimator (Chao 1) of bacterial taxa associated with *A. lobifera* across all three reef sites. (TIFF 480 kb)
