# Supplementary material for: Symbiosis and microbiome flexibility in calcifying benthic foraminifera of the Great Barrier Reef
Source: Microbiome. 2017 Mar 23;5:38. doi: 10.1186/s40168-017-0257-7 (PMC5364595; doi:10.1186/s40168-017-0257-7)
Supplement: Supplementary file 2 — Results of main effects PERMANOVA model of photosynthesizing eukaryotic taxa associated with Amphistegina lobifera collected from different reef sites. Table S4. Results of main effects PERMANOVA model and pairwise comparison of Bacillariophyta taxonomic composition in Amphistegina lobifera collected from different reef sites. Table S6. Results of main effects PERMANOVA model and pairwise comparison of bacterial community in Amphistegina lobifera collected from different reef sites. Table S7. Results of alpha diversity comparison between samples using a parametric t test using a t distribution. (DOC 58 kb) [file 40168_2017_257_MOESM2_ESM.doc]

**Symbiosis and microbiome flexibility in calcifying benthic Foraminifera of the Great Barrier Reef**

Martina Prazeres^1,*^, Tracy Ainsworth^2^, T. Edward Roberts^2^, John M. Pandolfi^2,3^, William Leggat^1,2^

^1^ College of Public Health, Medical and Veterinary Sciences, James Cook University, Townsville, QLD, Australia

^2^ARC Centre of Excellence for Coral Reef Studies, James Cook University, Townsville, QLD, Australia

^3^School of Biological Sciences, The University of Queensland, St. Lucia, QLD, Australia

*****Corresponding author:

Phone: + 61 7 4781 6009

Email: [martina.defreitasprazeres@jcu.edu.au](mailto:martina.defreitasprazeres@jcu.edu.au)

Table S2. Results of main effects PERMANOVA model of photosynthesizing eukaryotic taxa associated with *Amphistegina lobifera* collected from different reef

| **Source** | **df** | **SS** | **MS** | **Pseudo-F** | ***p*-value** |
| --- | --- | --- | --- | --- | --- |
| Reef sites | 2 | 19.38 | 8.70 | 2.65 | 0.02 |
| Residuals | 12 | 39.38 | 3.29 |  |  |
| Total | 14 | 56.77 |  |  |  |

sites.

| **Source** | **df** | **SS** | **MS** | **Pseudo-F** | ***p*-value** |
| --- | --- | --- | --- | --- | --- |
| **One-way PERMANOVA** |  |  |  |  |  |
| Reef sites | 2 | 3495 | 1797 | 11.95 | 0.0001 |
| Residuals | 12 | 1805 | 150 |  |  |
| Total | 14 | 5399 |  |  |  |
| **Pair-wise comparison** |  |  |  | t | ***p*-value** |
| Inner-shelf x Mid-shelf |  |  |  | 4.15 | 0.009 |
| Inner-shelf x Outer-shelf |  |  |  | 4.01 | 0.008 |
| Mid-shelf x Outer-shelf |  |  |  | 1.33 | 0.05 |

Table S4. Results of main effects PERMANOVA model and pairwise comparison of Bacillariophyta taxonomic composition in *Amphistegina lobifera* collected from different reef sites.

Table S6. Results of main effects PERMANOVA model and pairwise comparison of bacterial community in *Amphistegina lobifera* collected from different reef sites.

| **Source** | **df** | **SS** | **MS** | **Pseudo-F** | ***p*-value** |
| --- | --- | --- | --- | --- | --- |
| **One-way PERMANOVA** |  |  |  |  |  |
| Reef sites | 2 | 10623 | 5311 | 2.16 | 0.0001 |
| Residuals | 11 | 27102 | 2463 |  |  |
| Total | 13 | 37725 |  |  |  |
| **Pair-wise comparison** |  |  |  | **t** | ***p*-value** |
| Inner-shelf x Mid-shelf |  |  |  | 1.62 | 0.008 |
| Inner-shelf x Outer-shelf |  |  |  | 1.65 | 0.008 |
| Mid-shelf x Outer-shelf |  |  |  | 1.14 | 0.07 |

Table S7. Results of alpha diversity comparison between samples using a parametric *t*-test using a *t*-distribution.

| **Source** | **t** | ***p*-value** |
| --- | --- | --- |
| Inner-shelf x Mid-shelf | 1.08 | 0.93 |
| Inner-shelf x Outer-shelf | 0.89 | 1.00 |
| Mid-shelf x Outer-shelf | 2.07 | 0.23 |
