# Supplementary figures and images for: Symbiosis and microbiome flexibility in calcifying benthic foraminifera of the Great Barrier Reef
Source: Microbiome. 2017 Mar 23;5:38. doi: 10.1186/s40168-017-0257-7 (PMC5364595; doi:10.1186/s40168-017-0257-7)

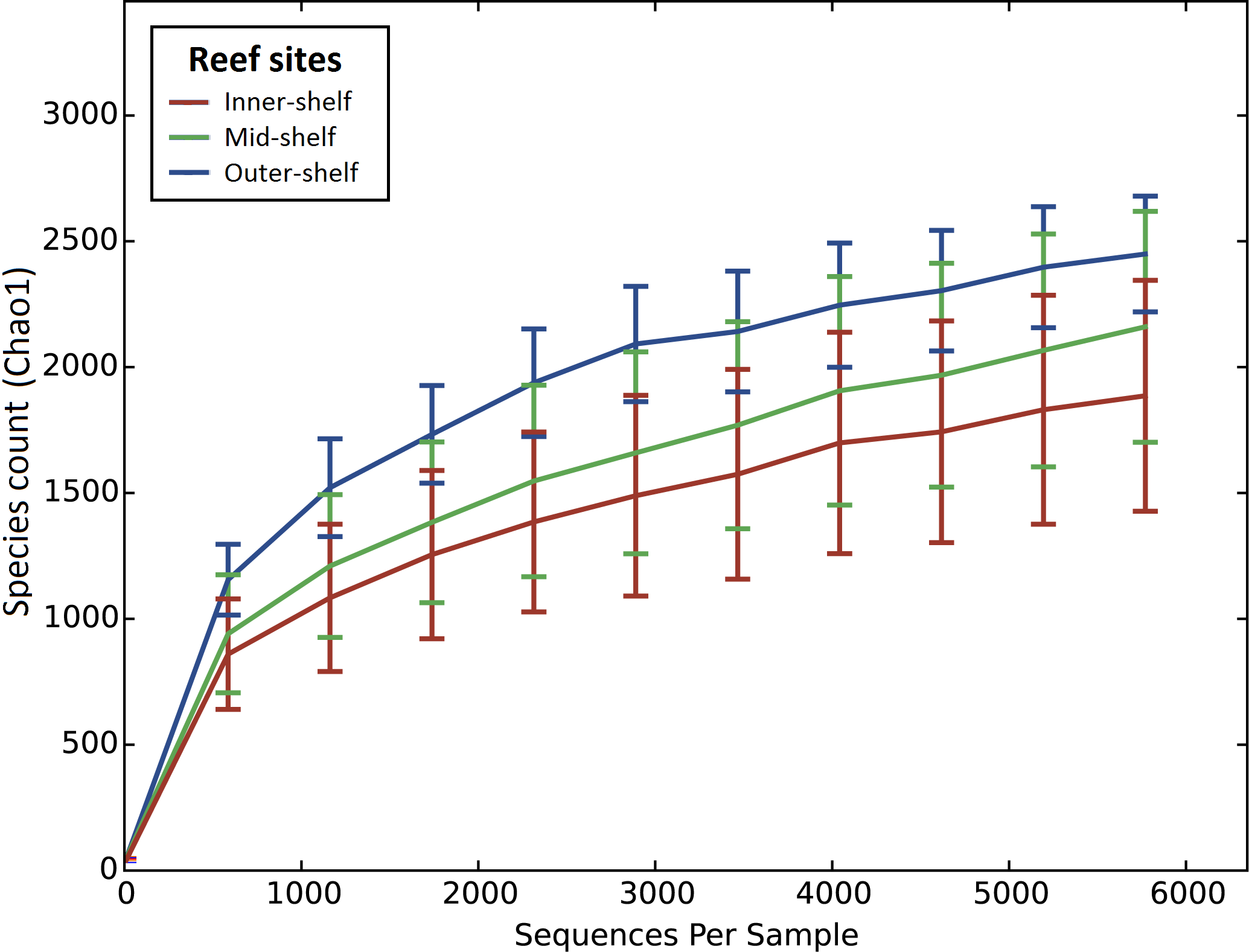

Supplement: Supplementary file 5 — Species richeness estimator (Chao 1) of bacterial taxa associated with A. lobifera across all three reef sites. (TIFF 480 kb) [file 40168_2017_257_MOESM5_ESM.tiff]
